# Exercise is not beneficial and may accelerate symptom onset in a mouse model of Huntington’s disease

**DOI:** 10.1371/currents.RRN1201

**Published:** 2010-12-07

**Authors:** Michelle C Potter, Chunyan Yuan, Conwell Ottenritter, Mohamed Mughal, Henriette van Praag

**Affiliations:** Laboratory of Neurosciences, National Institute on Aging, National Institutes of Health, Baltimore, MD 21224, U.S.A.

## Abstract

Exercise benefits both general health and brain function in rodents and humans. However, it is less clear whether physical activity prevents or ameliorates neurodegenerative diseases. The aim of the present study was to determine whether voluntary wheel running can delay the onset or reduce the severity of Huntington’s disease (HD) in a mouse model. To investigate whether running may delay HD symptoms lifespan, disease onset, locomotor activity, glucose levels, weight, striatal volume, inclusions, cognition and hippocampal neurogenesis were studied in male N171-82Q transgenic HD mice. Running started in pre-symptomatic (44±1 days old) male HD mice, did not improve function and appeared to accelerate disease onset. In particular, HD runners had an earlier onset of disease symptoms (shaking, hunched back and poor grooming), reduced striatal volume and impaired motor behavior, including a shorter latency to fall from the rotarod compared to sedentary controls. Furthermore, weight loss, reduced lifespan, hyperglycemia, Morris water maze learning deficits, diminished hippocampal neurogenesis, deficits in immature neuronal morphology, intranuclear inclusions and decreased dentate gyrus volume were refractory to physical activity. Taken together our research indicates that exercise is not beneficial, and may be detrimental to a vulnerable nervous system.

**Introduction**

      Exercise benefits general health and brain function [1].  In rodents, running increases hippocampal  neurogenesis, spine density, vascularization, neurotrophin levels, learning and long-term potentiation [2] [3] [4]. However, it remains unclear whether exercise delays or prevents neurodegenerative diseases and HD in particular. Initial research showed that environmental enrichment delays symptom onset in R6/1 transgenic mice [5]. An important component of enrichment is physical activity [6]. Indeed, in R6/1 mice, running normalized rearing behavior and delayed the onset of deficits in rear-paw clasping, motor coordination and spatial working memory. However, rotarod performance, ubiquitinated protein aggregates and hippocampal BDNF protein levels were unchanged by exercise [7] [8]. In addition, running conferred some positive changes to electrophysiological properties of medium-sized spiny neurons in the striatum of R6/2 mice [9]. Thus, it is unclear whether running enhances motor and cognitive function in HD mouse models.

      A biological correlate of exercise is increased hippocampal neurogenesis [4]. Stimulation of endogenous dentate gyrus neurogenesis [10] [11] may be a therapeutic intervention [12] for HD related cognitive deficits. However, in R6/2 mice, neurogenesis is reduced and refractory to exercise [13]. It is unknown whether lack of an effect of running is limited to this transgenic strain. R6/2 mice are mainly a model for the juvenile-onset form of HD, whereas R6/1 transgenic mice have a later disease onset and longer lifespan [14] [15]. Interestingly, environmental enrichment enhanced neurogenesis in this mouse model [16]. Effects of running on cell genesis in N171-82Q mice have not been investigated. 

      Transgenic N171-82Q mice express a human N-terminal truncated huntingtin cDNA encoding a 171 amino acid fragment with 82 polyglutamine repeats driven by a neuron specific mouse prion protein promoter [17] [18]. These mice have mutant Huntingtin-positive (mHtt^+^) nuclear inclusions, neuritic aggregates, motor and cognitive deficits, hyperglycemia, weight loss and reduced lifespan [15] [17] [18] [19] [20]. From 11 weeks of age, weight loss, tremors and clasping are found. Cognitive deficits begin around 14 weeks. By week 16, neuronal loss and mHtt^+^ inclusions are detectable in striatum, cortex and hippocampus. Total lifespan is 5-6 months [15] [17] [18] [19] [20]. The gradual symptom development allows for the study of this model of disease progression and for investigation of possible pre-symptomatic therapies.  

      In the present study, we aimed to determine whether HD can be delayed by starting voluntary exercise in pre-symptomatic N171-82Q mice. Surprisingly, running accelerated disease onset and exacerbated motor impairments and striatal volume reduction. In addition, physical activity did not improve hippocampal neurogenesis, dentate gyrus volume, weight, hyperglycemia, cognition, mHtt^+^ cell number and lifespan. Our research indicates that in a mouse model of HD, exercise is not beneficial, and could be detrimental for general health and brain function.    

**Materials and Methods**

**Subjects**

   WT C3H/HEJxC57BL/6J F_1_ hybrid females were mated with male transgenic N171-82Q mice expressing a human N-terminal truncated huntingtin with 82 polyglutamine repeats driven by a mouse prion protein promoter provided by D.R. Borchelt (Johns Hopkins University, Baltimore) [17] [18]. These animals were housed in a temperature-controlled animal facility on a 12 hour/12 hour light dark cycle with free access to food and water. The animal facility was fully accredited by the American Association for the Accreditation of Laboratory Animal Care (AAALAC).  The offspring were genotyped subsequent to weaning. In the lifespan mice (L, Fig 1A), the number of CAG repeats was sequenced (Table 1) in the transgenic animals (Laragen Inc. Los Angeles, CA).

**Figure:**
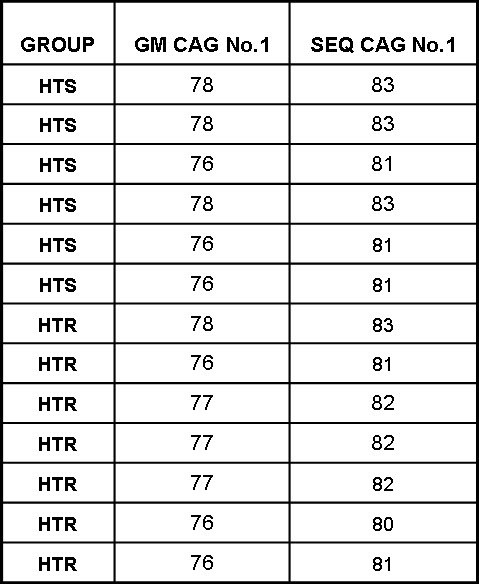
**Table 1. ** The number of CAG repeats in the HD mice used for lifespan (L) analysis. The CAG repeat number was measured by ABI Genemapper (GM) technique (Laragen Inc. Los Angeles, CA).  The CAG numbers were derived from the PCR fragment size. The DNA fragments with trinucleotide repeats such as CAG migrate faster than the DNA fragments with random sequences.   Therefore, the CAG repeat numbers determined by GM technique is smaller than the CAG repeat numbers determined by DNA sequencing technique (SEQ), which are closer to the actual CAG repeat numbers. Overall this analysis showed that CAG repeat number remained stable, had not expanded further with breeding and did not differ between transgenic controls and runners (p>0.59).  

**General Experimental Procedure**

      Male HD heterozygotes (N=26) and non-transgenic controls (N=28) (44±1 days old) were individually housed in standard cages with or without a running wheel (WTS=13; WTR=15; HTS=13; HTR=13) (Fig 1A). During the first 10 days of the experiment, all mice were injected with bromodeoxyuridine (BrdU, Sigma, St. Louis MO; 50 mg/kg) to label dividing cells. General health and disease symptoms, including body weight and glucose levels as well as running distance were recorded throughout the entire experiment in all mice. A subset (S) of these mice (S mice: WTS=6; WTR=5; HTS=7; HTR=6) were tested in the Morris water maze (88±1 days old), rotarod (100±1 days old) and open field (103±1 days old). On completion of behavioral testing (113±1 days old), these mice were deeply anesthetized with isofluorane (Abbott Laboratories) and perfused transcardially with 0.9% saline. One hemisphere was dissected and the cortex, hippocampus and striatum were stored at -80°C for future biochemical and molecular assays. The other hemisphere was placed in cold 4% paraformaldehyde (PFA) in 0.1 M PBS for 3-4 days, then transferred to 30% sucrose and processed for immunostaining as described below. The remaining animals were followed throughout their lives for the appearance of disease symptoms and lifespan comparisons (L mice: WTS=7; WTR=10; HTS=6; HTR=7). Upon development of severe symptoms resulting in immobility and inability to feed, transgenic mice were deeply anesthetized and perfused with 4% PFA in 0.1 M PBS and then equilibrated in 30% sucrose after 3-4 days. Once all the transgenic mice had died, the remaining WT mice were sacrificed and treated in the same manner. Sequential coronal sections (40 μm) were taken through the rostral-caudal extent of the brain and stored in phosphate-buffered glycerol at -20°C until further processing for immunostaining. For a timeline of the experiments see Fig 1A. 

**Health Assessment**

      General health assessments were performed biweekly. Mice (L) were checked for the presence or absence of symptoms including hunched back, poor grooming, involuntary shaking, erratic behavior, hind leg clinching, and inactivity (Table 2). The age of onset of disease symptoms was recorded as the first day any of the above mentioned symptoms were observed in each mouse (L mice). At regular intervals, body weight was recorded and glucose levels were measured using blood glucose test strips and a blood glucose meter (Ascensia ELITE XL Blood Glucose Meter). Weights and glucose measurements (S+L mice) in the study are presented at the age of acquisition of the measurement over the time course of the experiment.

**Table:** 

**GROUP**	**W 8**	**W 9**	**W 10**	**W 11**	**W 12**	**W 13**	**W 14**	**W 15**	**W 16**	**W 17**	**W 18**	**W 19**	**W 20**	**W 21**	**W 22**	**W 23**	**W 24**
**HTS**					G	GH	HSG	HSG	HSG	HSGC	HGS	HGS	HGS	HGS	HGS	HGS	HGSI
**HTS**						H	HS	S	HS	HSG	HSG	HSG	HSG				
**HTS**							SH	HSG	HSG	HSG							
**HTS**	H	H	H	S	HGS	HS	HSG	HSG	HSG	HSG	HSGC	HSG	HGSCI				
**HTS**			H	H	H	HSCG	HSG	HSG	HGSI								
**HTS**				H	GH	HS	HISG	HISG	HISG	HGS							
**HTR**		HS	H	H	HSB	GSH	HS	HSG	HGSC	HGS							
**HTR**		HS	H	H	HS	HG	HSGC	HSG	HSG	HSG	HSG	HSG	HSG	HSG	HSG	HSG	HGSC
**HTR**					H	HG	H	HSG	HSG	HSG	HSG	HGS	HGS	HGS	HGS	HGS	HGSI
**HTR**	HG	H	H	H	G	HS	HSG	HSG	HSG	HSG	HSG	HSG	HSG	HSG	HSG	HSG	HSGI
**HTR**			H	H	H	HSC	HSG	HSG	HSC	HSC	HGS	HGSI	HGSI	HGS	HGSI		
**HTR**	G	GS	HG	HG	HSG	HSGI											
**HTR**	S	HS	HS														

**Table 2.** The timing of the appearance of characteristic HD symptoms throughout the study following bi-weekly monitoring by the investigators. S = Involuntary shaking; C = Hind leg clinching; H = Hunched back; G = Poor grooming; I = Inactivity; B = Erratic behavior.  The age of onset of disease symptoms was recorded as the first day symptoms associated with HD were observed in each individual mouse in the lifespan group.

****

**Motor Skills**

Running wheel distance

      Running distances were recorded in kilometers weekly through a mini-computer (Sigma Sport, Batavia IL) attached to the running wheels. These data (S+L mice) are presented at the age of acquisition of the measurement over the time course of the experiment.

Rotarod tests

      An accelerating paradigm from 3 to 30 rpm was used for testing on the rotarod (S mice), with a maximum duration of 5 min per trial. The mice were placed on the rotating rod and the latency until they fell off and the number of falls over 5 min were recorded. This procedure was repeated 3 times consecutively and the averages were used as measures of locomotor ability. 

Open field

      Animals from the 4 groups (S mice) were tested in an open field arena (27.3 x 27.3 cm, height 20.3 cm) (Med Associates Inc., Georgia, VT). Each arena had a black floor and walls where x-y movements are monitored by two sets of pulsed-modulated infrared photobeams.   Animals were placed in the center of the arena at the beginning of the testing paradigm and were left undisturbed for 30 min. The total distance traveled in the open field over the 30 min was recorded and analyzed.

**Cognition**

Morris water maze

      Mice (S mice) were trained in the Morris water maze (pool diameter 1.4 m) for 8 consecutive days with 4 trials per day beginning at a different starting point every trial, as described previously [21] . Each trial was 1 min long with a 10 sec inter-trial interval. Once the mice found the platform, they were given a 15 sec rest on the platform. If the mouse was unable to find the platform in 1 min, it was manually placed on the platform for 15 sec. The latency to reach the platform and swim speed were recorded semi-automatically by a video tracking system (Anymaze, Stoelting Inc.). A 60 sec probe trial was performed 24 hours after the last training session. For this trial, the platform was removed and the mouse was allowed to swim for 60 sec. The time spent in each quadrant and the number of platform crossings were recorded.  

**Histological Analysis**

      Immunohistochemistry for BrdU and immunofluorescent double labeling for BrdU and NeuN were performed on separate free-floating one-in-six series of 40μm coronal sections (240μm apart) that were pretreated for BrdU immunohistochemistry by denaturing DNA, as described previously [21]. The primary antibodies used were BrdU anti-rat (1:200; Accurate, Harlan Sera-Lab) and mouse anti-NeuN (1:100; Millipore). To visualize BrdU-labeled cells for counting total number of BrdU^+^ cells, the peroxidase method was used (ABC system (Vector Laboratories, Burlingame, CA) with a biotinylated donkey anti-rat secondary antibody (Jackson Immunoresearch West Grove, PA) and diaminobenzadine (DAB) as a chromogen. For double-labeling experiments to determine the percentage of BrdU^+^/NeuN^+^ cells, the fluorescent secondary antibodies used were donkey anti-rat Alexa Fluor 488 (1:250; Invitrogen), and donkey anti-mouse Cy3 (1:250; Jackson ImmunoResearch, West Grove, PA). In addition, morphological assessment of the cells was carried out using doublecortin (DCX) staining. Specifically, a 1:12 series of sections were labeled with DCX (1:250; Santa Cruz, CA) and the fluorescent secondary antibody used was donkey anti-goat Cy5 (1:250; Jackson ImmunoResearch, West Grove, PA). Complexity of dendritic branching of DCX-labeled immature neurons was assessed by an investigator blinded to the group identity.

      BrdU^+^ cell counts were performed through a 20X objective (Olympus BX51) throughout the entire GCL and hilus of the DG by a blinded investigator, as described [21]. An adjacent one-in-six series of sections were stained with DAPI (4’-6-diaminodino-2-phenylindole, 1 μl/10 ml Tris-buffered saline (TBS) for 10 min) to estimate GCL total volume. The GCL area was traced with a 20X objective using stereology software (StereoInvestigator, MicroBrightfield). The GCL volume was determined by the sum of the traced areas from the one-in-six series of sections multiplied by the distance between sections (240μm). The number of BrdU-labeled cells was matched to GCL volume and multiplied by the reference volume to estimate total number and density of BrdU^+^ cells in the GCL.

      To investigate the phenotype of these newly born cells an additional one-in-six series of sections were double-labeled with BrdU and NeuN as described above and visualized by confocal microscopy (Fluoview, Olympus). At least 50 BrdU^+^ cells per animal were analyzed for the co-expression of BrdU and NeuN. The percent of total BrdU^+^ cells co-labeling with NeuN were determined (% BrdU^+^/NeuN^+^ cells). Based on this percentage, the total number of BrdU^+^/NeuN^+^ cell numbers could be calculated from the total number of BrdU^+^ cells.  Striatal volume was measured in the same tissue used to perform the BrdU^+^ cell counts. Briefly, the average of the total area of striatal tissue from 3-4 sections prior to the anterior commissure was used for striatal volume. The percent change from control mice (WTS) was calculated and used for statistical analysis.

      Immunohistochemistry for mHtt was performed on separate free-floating one-in-twelve series of 40μm coronal sections (480μm apart) The primary antibody used was mHtt anti-mouse (1:100 Millipore, CA) To visualize mHtt-labeled cells, the peroxidase method was used (ABC system (Vector Laboratories, Burlingame, CA) with a biotinylated donkey anti-mouse secondary antibody (Jackson Immunoresearch West Grove, PA) and diaminobenzadine (DAB) as a chromogen. The optical fractionator method (sampling grid = 75x75μm) (Stereoinvestigator, Microbrightfield, VT) was used to quantify the number of cells with mHtt^+ ^ inclusions in 2 representative sections by a blinded investigator with a 40X oil objective (Olympus BX51). The estimated number of mHtt^+^ cells was matched to the traced GCL area and multiplied by the reference volume to estimate the number of mHtt^+^ cells.

**Statistical Analysis**

      For weights, glucose and running distance, a mixed effects statistical model was used to estimate the longitudinal trajectories while accounting for the within-subject correlations [22] . Weights, glucose and running distance were the dependent variables and the predictors included group and time. All the analyses were conducted in R (version 2.9.0). All other statistical analysis was carried out using Statview (Abacus Corporation) unless otherwise stated. For the Morris water maze, latency and swim speed, a two way ANOVA (Genotype x Exercise) with repeated measures (Time) was performed followed by a one-way ANOVA and Fisher’s post-hoc tests for individual days. A power analysis was performed for comparing four groups simultaneously and the statistical power obtained was 0.80 (SAS). For histological, rotarod, open field and Morris water maze probe trial data, a two way ANOVA (Genotype x Exercise) was performed followed by Fisher’s post-hoc tests. Since the HD mice did not cross the platform zone during the probe trial an unpaired student's t-test was performed to compare the WTR and WTS groups. For striatal volume measurements, a two way ANOVA (Genotype x Exercise) was performed followed by post-hoc t-tests. For mHtt^+^ cell number and disease onset data, unpaired student’s t-tests were carried out. 

**Results**

**Running accelerates the age of onset and increases the severity of HD symptoms**

      The age of onset of disease was recorded as the first day symptoms associated with HD were observed in bi-weekly assessments in the L mice (Fig 1A,B). It was observed that running significantly accelerated onset of disease symptoms (t_(11)_=2.44: p<0.03). HTR mice presented with symptoms such as hunched back, poor grooming and involuntary shaking an average of 16 days (±2.17 days) sooner than sedentary (HTS) controls (Fig 1B & Table 2). Although running accelerated disease onset, it did not influence lifespan in HD mice (t_(11)_=0.20: p<0.84; Fig 1C). All HD mice had died by 26 weeks of age (Fig 1A,E).

**Figure:**
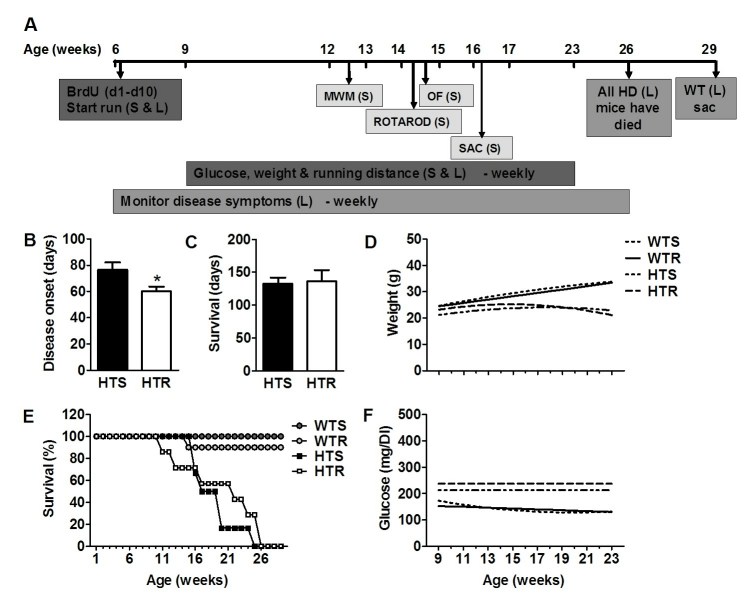
**Figure 1.** Running accelerated the onset of characteristic HD disease symptoms but did not affect hyperglycemia, weight loss or lifespan. (A) Timeline of experiment in weeks of age in the subset (S) and lifespan (L) mice (WT, N=28; HD, N=26, at the beginning of the study). Behavioral testing was carried out from week 12 to 15 (S mice: WTS=6; WTR=5; HTS=7; HTR=6). Mice (S) were sacrificed (SAC) at approximately 16.2 weeks of age. Survival analysis (L mice: HTS=6; HTR=7) indicated that HD mice died between 11 and 26 weeks of age. WT controls (L mice: WTS=7; WTR=10) were sacrificed at 29 weeks. Abbreviations: MWM =Morris water maze; OF= Open field; S=Subset; L= Lifespan group; SAC = sacrificed. (B) Onset of disease symptoms (L mice) such as hunched back, poor grooming and involuntary shaking occurred earlier in HD runners; * p<0.03. HTR (N=7) versus HTS (N=6). Data represents mean ± SEM (C) Lifespan (L mice) did not change as a result of exercise. Data represents mean ± SEM. (D) Body weight (S+L) was reduced in HD mice. There were significant differences in time-related change for WTR vs HTR (p<0.0001) and WTS vs HTS (p<0.01). (E) Survival plot indicating the rate of attrition (S+L). (F) HD mice had increased blood glucose levels compared to WT controls. There were significant differences in time-related change for WTR vs HTR (p<0.0003) WTS and HTS (p<0.05).

      HD mice display progressive weight loss or failure to continue to gain weight over their lifetime [15] [17] [18] [19] [20]. Weight measurements were taken for every mouse (WTS=13; WTR=15; HTS=13; HTR=13) up to week 11, after which time mice were progressively lost from the study (Fig 1E). Therefore, a mixed effects statistical model was used to estimate the longitudinal trajectories while accounting for the within-subject correlations. There were significant differences in time-related change for WTR vs HTR (F_(2,44.1)_ = 20.36, p<0.0001) and WTS vs HTS (F_(2,52.1)_ = 4.54, p<0.01) (Fig 1D). The differences for WTR vs WTS and HTR vs HTS were not statistically different.

      Both HD patients and mouse models of the disease appear to show deficient glucose metabolism [18] [23] [24] [25]. In our study, similar statistical analysis was performed as described above for weights. There were significant differences in time-related change for WTR vs HTR (F_(2,82.5)_ = 9.07, p<0.0003) and WTS and HTS (F_(2,79.2)_ = 3.04, p<0.05) (Fig 1F). The differences for WTR vs WTS and HTR vs HTS were not statistically different. 

**Running increases the severity of motor deficits**

      Deficits in locomotor activity are a hallmark of HD [15] [17] [18] [19] [20]. We investigated the effect of running on rotarod performance (100±1 days old)  and open field behavior (103±1 days old) (S mice). In addition, running distance (Fig 1A) and striatal volume were measured in both groups (S and L mice).

      The latency to the first fall from the rotarod and the number of falls over 5 min was recorded in three consecutive trials and the average of these three trials was calculated (Fig 2 A,D). There was a significant main effect of genotype in the latency to first fall (F_(1,20)_ = 4.56, p<0.05) and for the number of falls (F_(1,20)_ = 5.99, p<0.02). Specific comparisons showed that the HTR mice fell off the rotarod sooner than the other three groups (p<0.03; Fig 2A) and had more total falls than the two WT groups (p<0.02; Fig 2D).  

**Figure:**
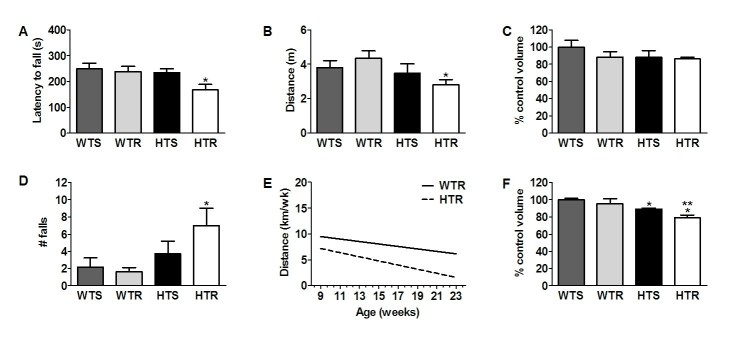
**Figure 2.** Exercise exacerbated locomotor deficits in HD mice and reduced striatal volume. (A,D) The latency to fall off an accelerating rotarod was shorter in HTR mice than in all other groups (A); * p<0.03 (S mice: WTS=6; WTR=5; HTS=7; HTR=6). The total number of falls from the rotarod over 5 min was increased in HD runners compared to WT mice (D); * p<0.02, HTR versus WTS & WTR. (B) The total distance travelled over 30 min in an open field was reduced in HD mice compared to WT mice, specifically in HTR mice compared to WTR mice; * p<0.03, HTR versus WTR (S mice: WTS=6; WTR=5; HTS=7; HTR=6). (C,F) The striatal volume (% WTS control volume) was not significantly different between groups in the S mice (C). In the L mice transgenic runners had a significantly diminished striatal volume; **p<0.05, HTR versus HTS. In addition, both HTS and HTR (L) differed significantly from WTS (*p<0.006) (F). (E) There was no significant difference between the groups in running distance over the duration of the experiment (p>0.16). Data represents mean ± SEM.

      Analysis of distance traveled in the open field over 30 min only revealed a significant main effect of genotype (F_(1,20)_ =4.39, p<0.049; Fig 2B). Overall, HD mice had reduced open field activity as compared to WT controls. Posthoc tests showed that the HTR covered significantly less distance in the open field than WT running mice (WTR) (p<0.03: Fig 2B). 

      Voluntary running distance in the home cage was monitored in WTR and HTR groups and data were analyzed as described for weights and glucose above. There was no significant difference between the groups in running distance over the duration of the experiment (F_(2,275)_ = 1.83, p < 0.16; Fig 2E).

      Striatal volume was measured in both S and L groups.  Analysis of the percent change in volume in the HD mice compared to WT control mice showed no difference between the groups in the S mice (Fig 2C), but did reveal a significant main effect of genotype for the L group (F_(1,20)_ =6.24, p<0.02); Fig 2F). Posthoc tests showed that HTR  mice (L) had significantly diminished striatal volume compared to HTS mice (p<0.046: Fig 2F), and both groups differed significantly from WTS mice (p<0.006: Fig 2F). 

**Running fails to rescue cognitive impairment**

      Morris water maze [26] performance was tested around the time when cognitive abnormalities appear (88±1 days old; S mice; Fig 1A) [19]. Mice were trained over 8 days with 4 daily consecutive trials. A two-way ANOVA with repeated measures (Days) revealed a significant difference in latency to the platform, with main effects of genotype (F_(1,20)_=74.08, p<0.0001) and exercise (F_(1,20)_ = 4.88, p<0.04), (Fig 3A). There was no interaction between genotype and exercise (p>0.39). WT mice performed better than the transgenic animals (p<0.0002). Furthermore, WTR had a greater rate of learning on this task than all other groups (p<0.048). Learning curve comparison revealed a significant decrease in latency from day 1 to day 8 in the WT mice (F_(1,7)_ =5.36, p<0.0001) but not in the transgenic mice (F_(1,7)_ =0.64, p>0.72). Swim speed analysis showed main effects of genotype (F_(1,20)_ = 28.25, p<0.0001) and exercise (F_(1,20)_=5.04, p<0.04), and no interaction (p>0.29), (Fig 3B). Overall transgenic mice swam slower than the WT mice (p<0.04). However, the HTR mice swam faster than the HTS mice (p<0.02). There was no difference in swim speed between WT sedentary mice (WTS) and WTR (p>0.44), suggesting enhanced WTR performance is not due to improved motor skills.

**Figure:**
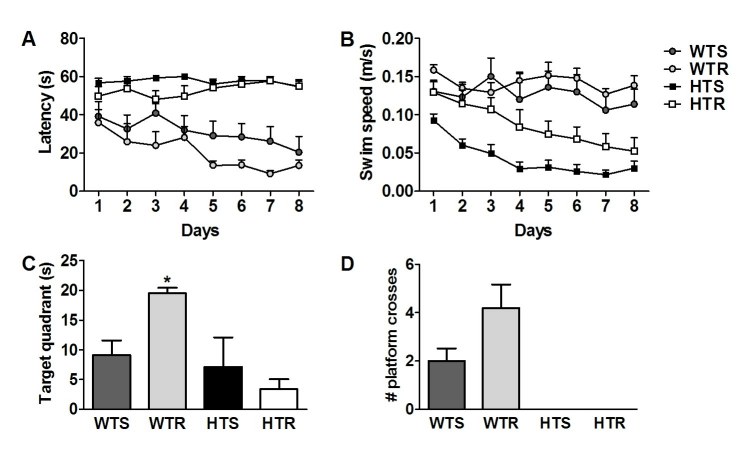
**Figure 3.** Running did not rescue the spatial learning deficit in the Morris water maze. (A) Latency to reach the platform in the Morris water maze over 8 test days (data represents the average of 4 trials per day) was reduced in WT  but not HD  mice; p<0.05, (S mice: WTS=6; WTR=5; HTS=7; HTR=6). WTR mice had a greater rate of learning on this task than all other groups; p<0.05. (B) Swim speed was slower in HD than WT mice; p<0.05. (C) A probe trial 24 hr after the last training trial on day 8 showed that WT runners preferred the platform quadrant significantly more than the other groups; * p <0.02, WTR versus WTS, HTS & HTR. (D) Platform crosses were not significantly different between WTR and WTS groups (p>0.06) . HD mice did not cross the platform zone during the probe trial. Data represents mean ± SEM.

      A probe trial was performed 24 hr after the last training session. For time spent in the target quadrant there was a significant genotype x exercise interaction (F_(1,20)_ = 4.53, p<0.05). Specific comparisons showed that only the WTR group spent more time in the target quadrant compared to the three other groups (p<0.05) (Fig 3C). HD mice did not cross the platform zone during the probe trial. There was no significant difference in the number of platform crossings between WTR and WTS mice (p>0.06) (Fig 3D). 

**Running does not reverse decreased neurogenesis or diminish mHtt inclusions**

      Neurogenesis is decreased in mouse HD models [13] [16] [27] [28] [29]. Data for all mice tested (S and L mice; Fig 1A) did not differ and was therefore combined (WTS=13; WTR=15; HTS=9-12; HTR=10-12). There was a significant main effect of genotype on BrdU^+^ cell number (F_(1,48)_ = 41.14, p<0.0001) and density F_(1,48)_ = 33.52, p<0.0001). HD mice had reduced BrdU^+^ cell number and density compared to WT animals (p<0.002). WTR had more BrdU^+^ cells than all the other groups (p<0.03) confirming that running enhances new cell survival under standard conditions, whereas there was no effect of exercise in HD mice (p>0.93) (Fig 4A,B). To identify the percentage of surviving BrdU^+^ cells that become neurons, double labeling for BrdU and NeuN was performed.  Significant main effects of genotype (F _(1,43)_ = 303.37, p<0.0001) and exercise (F_(1,43)_ = 7.89, p<0.008) and no interaction of genotype and exercise (p>0.72) were observed. The percentage of double-labeled BrdU^+^/NeuN^+^ cells was significantly higher in HTR than HTS mice (p<0.05; Fig 4C). However, the total number of new neurons did not differ between the HTR and HTS groups (p>0.88; Fig 4D). Furthermore, analysis of granule cell layer (GCL) volume revealed a main effect of genotype (F_(1,48)_ =18.29, p<0.0001; Fig 4E), suggesting that reduced neurogenesis in HD mice may have translated to a significant volume reduction. Alternatively, this volume reduction could be explained by neuronal atrophy leading to reductions in hippocampal volume [30].  

**Figure:**
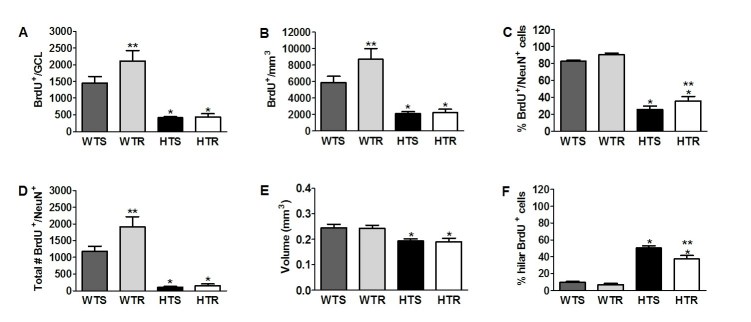
**Figure 4. **Exercise failed to rescue the neurogenic deficit and decreased GCL volume in HD mice. (A) The number of BrdU^+^ cells was significantly increased in WTR as compared to all other groups; ** p<0.03 (S+L mice; WTS=13; WTR=15; HTS=12; HTR=12). HD mice (N=24) had significantly less cells than WT (N=28); *p<0.002. (B) The density of BrdU^+^ cells (BrdU^+^/mm^3^) was highest in WTR, ** p<0.02, and significantly reduced in HD mice compared to WT mice, * p<0.006. (C) The percentage of BrdU^+^/NeuN^+^ was greater in WT than HD mice, ** p<0.0001, and in HTR as compared to HTS mice; p<0.05.  (D) The total number of new neurons was significantly increased in WTR, ** p <0.01 versus all other groups, as well as in WT as compared to HD mice; * p<0.002. (E) GCL volume measurements showed a reduction in HD versus WT mice; * p<0.005. (F) The percentage of BrdU^+^ cells present in the hilus was less in WT than HD mice, * p<0.0001, and also less in HTR than in HTS mice; ** p<0.0007.  Data represents mean ± SEM.

      An interesting observation was made pertaining to the location of BrdU^+^ cells. There was a higher percentage of hilar BrdU^+^ cells in HD compared to WT mice (F_(1,43)_=242.56, p<0.0001). There was also a significant interaction between genotype and exercise (F_(1,43)_=4.82, p<0.03). Specific comparisons showed that HTR had a significantly lower percentage of hilar BrdU^+^ cells than HTS (p<0.0007; Fig 4F). Together with the increase in percentage BrdU^+^/NeuN^+ ^cells, this suggests a possible modest beneficial effect of exercise on neurogenesis in HTR mice. However, doublecortin (DCX) staining revealed that the immature neurons in both HD groups appear to have less dendritic branching and dendrites that do not extend into the molecular layer as compared to WT mice (Fig 5).

**Figure:**
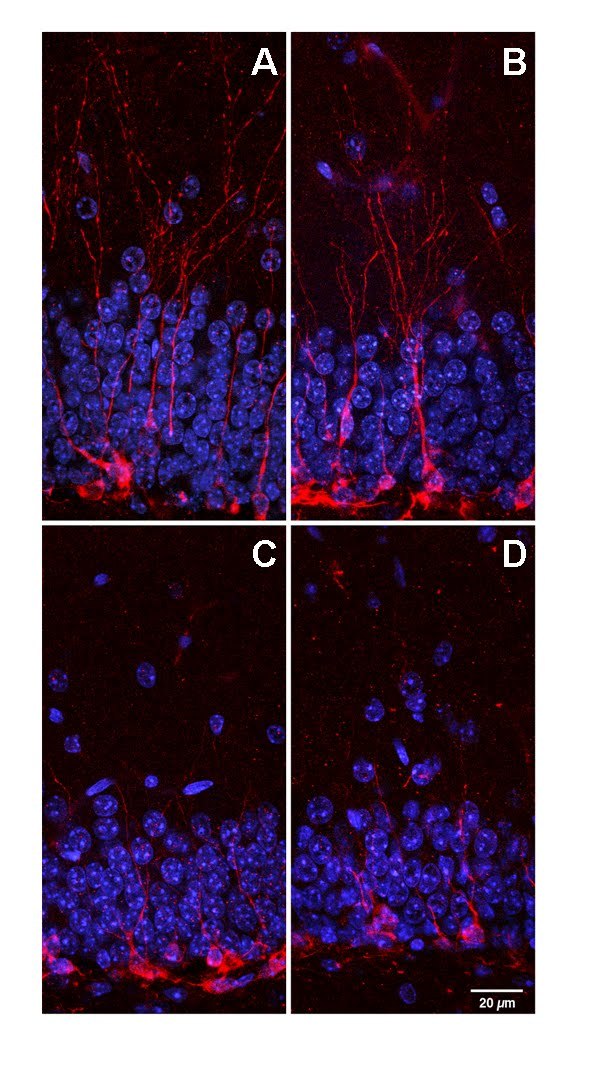
**Figure 5.** Morphology of Doublecortin (DCX) expressing immature neurons in the dentate gyrus of WT and HD mice. Dendritic branching complexity of DCX-labeled (red) immature neurons was reduced in the transgenic mice, (A) WTS, (B) WTR, (C) HTS and (D) HTR. Granular cell layer neurons were labeled with DAPI (blue). Scale bar = 20μm.  

      To further investigate the effects of physical activity in this disease model, cells with mHtt^+^ inclusion bodies were quantified in the granule cell layer in the mice (S) who were sacrificed at an average of 113±1 days old [15] [20] [31]. Running had no effect on the number of cells with mHtt^+^ inclusion bodies in the dentate gyrus of the hippocampus (Fig 6).  

**Figure:**
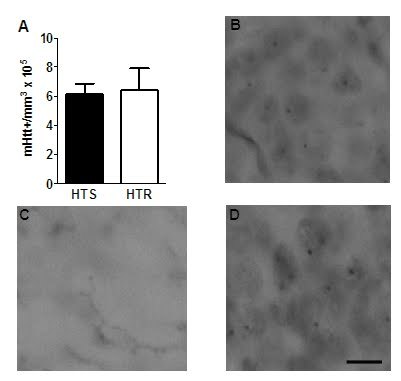
**Figure 6. **Running does not alter the number of DG cells with mHtt+ inclusions. (A) The number of cells with mHtt+ inclusions (mHtt^+^/mm^3^) does not differ between HTR and HTS mice. Data represents mean ± SEM (HTS=7; HTR=6). Representative photomicrograph showing hippocampal mHtt^+ ^ staining in HTR (B) WTS (C) HTS (D). Scale bar = 10µm.   

**Discussion**

      It is assumed that exercise can delay, prevent or ameliorate neurodegenerative disease [1] largely because of its profound benefits under standard conditions [1] [4]. However, the present study shows that exercise impaired motor performance and reduced striatal volume and age of disease onset in a HD mouse model. In addition, total lifespan, progressive weight loss, hyperglycemia, reduced neurogenesis, deficits in immature neuronal morphology, intranuclear inclusions, decreased GCL volume and impaired cognitive performance were not changed by exercise. Our findings differ from previous investigations with R6/1 mice [7] [8]. While it could be considered that our results are model-specific, this is unlikely. Previous work in R6/2 mice showed that exercise did not increase neurogenesis [13], similar to our findings in the N171-82Q mice. In addition, treatment with the antidepressant sertraline improved symptoms and neurogenesis in both the R6/1 and N171-82Q models [32] [33], indicating similar efficacy of this type of intervention in both models. Furthermore, in the studies where some benefit of exercise was reported in R6/1 mice [7] [8], the animals were group-housed, making it unclear how much and whether individual mice ran. In our study, the mice were singly housed to obtain accurate running distance measures and because recent research indicated that stress and neurogenesis levels were not influenced by housing [34]. Moreover it is reported that R6/1 running mice travel less distance in the open field than sedentary transgenic mice [7]. This was interpreted as a lack of interest in the open field arena [7] rather than as an emerging motor deficit. Finally, a statistical power analysis was carried out which further supports the validity of our data. 

      Although we show that exercise had a negative impact on multiple HD symptoms, other interventions such as dietary restriction and environmental enrichment do appear to delay HD symptoms and increase survival rates in HD mouse models [5] [18] [35] [36]. In particular, environmental enrichment may have a superior outcome compared to running.  Possibly, non-strenuous elements of enrichment, such as increased social interaction and nesting materials may mediate the benefits. Voluntary wheel running can be considered as a controllable voluntary stressor [37]. Applying physical exercise to a mouse genetically predisposed to develop HD may have caused unwelcome stress to an already vulnerable system [38]. Further impairments in the minimal spatial learning and neurogenesis levels may not be possible, but the exacerbation of motor symptoms and acceleration of disease onset may have resulted. Indeed, stress may hasten the appearance of certain neurodegenerative diseases or other pathologies [39] [40] [41] [42]. It has been shown that exercise may have detrimental effects following stroke [43]. Moreover, the energetic demands of exercise in this metabolically-challenged transgenic mouse [18] may further the negative impact of running by possibly releasing as of yet unidentified aberrant factors into the bloodstream that could lead to functional impairments. 

      In the R6/1 mice, running has been shown to normalize rearing behavior and delay a deficit in rear-paw clasping and motor coordination on a static rod but did not improve rotarod performance [7] [8]. In our study, the accelerating rotarod revealed motor deficits only in the HD runners in the latency to fall. It is possible that running resulted in earlier striatal atrophy. Although a change in striatal volume in HD runners was not observed in mice used for the motor behavioral tests (S), it was reduced in the lifespan group (L). This raises the possibility of a subtle, but detrimental effect of exercise that translated to the motor impairments. While this is the first animal study making observations that exercise may not be beneficial in HD, there is evidence from human studies that points in the same direction. Recently, a study in humans indicated that physical exercise may not prevent or delay disease onset or progression [44] [45]. A case study of a HD marathon runner presented with myopathy years before the predicted disease onset. In fact, this patient had onset of HD symptoms almost twenty years before the median age of onset for 41 CAG repeats, indicating that regular exercise certainly did not delay the disease development [44] [45]. Indeed, prescription of exercise protocols to patients with neurodegenerative diseases should be done with caution [46].

        Huntington’s disease is often defined as affecting the striatum and cortex [15] [19] [20]. However, the hippocampus also shows profound neuronal loss in HD patients and animal models [13] [27] [28] [29] [47] [48] [49]. In the present study, we observed a decrease in hippocampal volume and neurogenesis. Exercise did not reverse these deficits in N171-82Q mice similar to previous research in R6/2 mice [13], nor was the number of cells with mHtt^+^ intranuclear inclusions changed. In addition, consistent with R6/2 mice, a significant increase in the percentage of newborn cells that took on a mature neuronal phenotype was observed but this did not translate to an increase in total neuron number [13]. Furthermore, a reduced percentage of hilar BrdU^+^ cells was observed in HD runners as compared to sedentary controls. While this could be considered a positive effect of running, the immature neuronal marker doublecortin showed that cells did not extend processes into the molecular layer, suggesting neurite extension and maturation deficits in both groups of transgenic mice. Likely, the hippocampal microenvironment plays an important role [50]. Indeed, the exercise-induced survival factor BDNF[2] [51] is reduced in HD mice and not increased by running [7]. Administration of this factor did enhance striatal neurogenesis [52] and may have a similar effect in the hippocampus.

      Running improves performance in spatial memory tasks under normal physiological conditions. This is thought to be mediated, at least in part by increased synaptic plasticity and neurogenesis [4]. In our study, we found no improvement in HD mice as a result of running in spatial learning in the Morris water maze, correlating with the severe reduction in adult neurogenesis. One possible confounding factor in our behavioral assessment of spatial memory was that the HD mice displayed a reduced swim speed, possibly due to the onset of motor impairments [15] [19] [20]. However, further evidence that the poor performance was indeed a cognitive deficit was that the HD mice did not have any learning curve, even at reduced speed. The HD mice did not differ in their latency to find the hidden platform from day 1 to day 8 indicating that they could not learn the task.  In addition, in the probe trial they showed no preference for the platform quadrant. WT runners had the best performance, as expected [4] [21]. It is possible that the water maze task is not ideal to test the possible benefit of therapeutic interventions in HD mice. Further research using memory tasks with low motor demand [53] [54] may be better suited to test cognition in these mouse models.

      It is clear from our study that running did not prevent or delay symptoms of HD but appeared to worsen the outcome in HD mice. Moreover, cognitive and neurogenic deficits were not reversed by running. Taken together, our research suggests that exercise is not beneficial and may even be detrimental in this animal model.  

**Acknowledgements**

      We thank Linda R. Kitabayashi for help with figure preparation.  We thank Dr. Bronwen Martin for helpful advice, and Dr. Tali Kobilo, David Creer, Kriti Gandhi and Robert Rowe for assistance with histology. Statistical assistance was kindly provided by Dr. Susan Resnick, Dr. Yang An and Dr. Larry Brant.

**Funding**

This research was supported by the Intramural Research Program at the National Institute on Aging.**** **Competing interests ** The authors have declared that no competing interests exist.  
